# Therapeutic monitoring of anti-seizure medications in low- and middle-income countries: a systematic review

**DOI:** 10.12688/wellcomeopenres.16749.1

**Published:** 2021-04-29

**Authors:** Mercy Odhiambo, Symon M. Kariuki, Charles R. Newton

**Affiliations:** 1 Neurosciences Unit, KEMRI Wellcome Trust Research Programme, KILIFI, 80108, Kenya; 2 Department of Public Health, School of Human and Health Sciences,, Pwani University,, KILIFI, 80108, Kenya; 3 Department of Psychiatry, University of Oxford, Oxford, UK

**Keywords:** Therapeutic drug monitoring, TDM, anti-seizure medication, ASM, low- and middle-income countries, LMICs, resource-limited settings

## Abstract

**Background**: The treatment gap for epilepsy is large in low- and middle-income countries (LMICs) and the effectiveness and safety of the available anti-seizure medication (ASMs) is not fully understood. We conducted a systematic review to evaluate the extent of therapeutic drug monitoring (TDM) of ASM in LMIC, including purposes and methods used during monitoring.

**Methods**: A search was conducted on four main databases (PubMed, Psych-Info, CINAHL and Embase), with eligible articles screened using a PRISMA checklist and a set of exclusion and inclusion criteria. Full texts were examined to evaluate the extent and practice of TDM in LMICs. Descriptive statistics were used to pool median distribution of TDM across studies.

**Results**: Of the 6,309 articles identified in the initial search, 65 (1.0%) met the eligibility criteria. TDM of ASMs was mostly done to assess toxicity (42.8%), but rarely to monitor adherence (9.0%). TDM differed by economic status and infrastructural status with majority of the studies coming from Europe (53.8%) and upper-middle-income countries (87.6%). First generation ASMs (82.3%) were more likely to be monitored than second generation ASMs (17.6%) and carbamazepine was the most frequently monitored drug. Fluorescence Polarization Immunoassay (FPIA) was the most common technique used for TDM (41.5%) followed by High-Performance Liquid Chromatography (HPLC) (16.9%). In addition, FPIA was the cheapest method of TDM based on approximated costs ($1000, TDx system). Assay validation and quality control were reported variably, and reference ranges used during TDM of ASMs were relatively uniform.

**Conclusions**: TDM is mostly done to evaluate ASM toxicity, but rarely for other reasons such as evaluating adherence or assessing clinical efficacy. There is a need for more investment in comprehensive TDM in LMICs that incorporates dose titration of ASM using pharmacokinetics and pharmacodynamics modelling, for both first generation and second generation ASMs.

## Introduction

Epilepsy is a serious neurological disorder characterized by an enduring predisposition to experience unprovoked seizures and often results in neurobehavioural, psychosocial consequences ( Fisher *et al.*, 2005) and premature mortality ( Levira *et al.*, 2017). Approximately 70 million people worldwide have epilepsy, more than 85% of whom live in low- and middle-income countries (LMICs) ( Ngugi *et al.*, 2011; World Health Organization, 2018). Appropriate treatment schedules of anti-seizure medication (ASMs), the main-stay of therapy for seizure disorders, results in complete control of seizures in up to 70% of patients yet the treatment gap is still large in LMICs ( Mbuba *et al.*, 2012; Meyer *et al.*, 2010). In these resource-poor settings, health-seeking behavior depends on availability and accessibility of health care facilities, cultural beliefs and financial resources ( Newton & Garcia, 2012), resulting in people seeking treatment only once the disease is already severe ( Kariuki *et al.*, 2015).

The ASMs such as carbamazepine, phenobarbital, phenytoin and valproic acid are listed in the Health Organization (WHO) essential medicines list as first line anti-seizure medications for the management of epilepsy ( WHO Model List of Essential Medicines, 2020). Lamotrigine and ethosuximide are recommended for the management of focal and focal to bilateral tonic-clonic seizures and absence seizures, respectively. Benzodiazepines such as clonazepam and diazepam are commonly used in resource-limited countries for acute management of acute seizures including status epilepticus ( World Health Organization, 2004). Phenobarbital, phenytoin, carbamazepine and sodium valproate are widely available in public health facilities in LMICs ( Ibinda *et al.*, 2017), while second generation ASMs such as ethosuximide, gabapentin, lamotrigine, topiramate, levetiracetam, pregabalin and clobazam are only available in private facilities or tertiary centres, not within the reach of the poor populations ( Chin, 2012).

ASM therapy requires a careful balance of controlling seizures while minimizing toxicity. Routine monitoring allows the prescriber to check treatment efficacy while identifying adverse effects and drug interactions. Drug interactions are often complicated by pharmacogenetics, medication for comorbidities and patient adherence ( Knezevic & Marzinke, 2018). For instance, phenobarbital is associated with adverse effects including risk of withdrawal seizures, hyperactivity in children, risk of drug abuse and suicidal ideation ( Nimaga *et al.*, 2002), but it is the only ASM that is easily available and affordable in many LMICs ( Chin, 2012). These adverse effects require monitoring but TDM is rarely done in LMICs due to limited infrastructure, resources and lack of technical expertise to operate equipment for mass spectrometry and immunoassays ( Tomson *et al.*, 2007). In view of the gaps in availability, access and effects of use of ASMs in LMIC highlighted above, we conducted a review to examine the extent of TDM, purposes and methods used during monitoring in these settings.

## Methods

### Inclusion and exclusion criteria for selecting studies

This review focused on studies reporting monitoring of both detectable and optimal levels of all ASMs in human serum, plasma and other body fluids. We included only primary studies originating from low-, lower-middle or upper-middle-income countries using the World Bank GNI per capita cut-offs for the 2021 fiscal year ( World Bank, 2020). The studies included were either cross-sectional, retrospective or prospective cohort studies published in peer-reviewed journals. Only articles written in English were included and there was no year of publication restriction. Reviews, case studies, conference abstracts, poster sessions, editorials and case reports were excluded from this review.

### Search strategy

The main search terms “monitoring”, “antiepileptic drugs” and “low- and middle-income countries” were developed, connected with a Boolean operator and then entered into the major databases ( Table 1). The initial database search was carried out on 8 ^th^ November 2018 and later updated to 31 ^st^ December 2020. The results in different databases were as follows: PubMed (N= 2,964), Psych-Info (N= 86), CINAHL (N= 39) and Embase (N= 3,821).

**Table 1.** Search strategy.

((Monitoring) OR (Measurement) OR (Evaluation) OR (Examination) OR (Observation) OR (Concentration) OR (Levels)) AND ((Antiepileptic drugs) OR (AED) OR (Antiepileptic agents) OR (Antiseizure medications) OR (ASM) OR (Anticonvulsants) OR (Carbamazepine) OR (Phenobarbital) OR (Phenytoin) OR (Sodium Valproate) OR (Ethosuximide) OR (Levetiracetam) OR (Gabapentin) OR (Lamotrigine) OR (Topiramate) OR (Pregabalin) OR (Clobazam) OR (Diazepam) OR (Clonazepam)) AND ((LMICs) OR (Low-income countries) OR (Lower- middle-income countries) OR (Upper-middle-income countries) OR (Sub-Saharan Africa) OR (Africa )OR (East Asia) OR (South Asia) OR (Middle East) OR (South America) OR (Tajikistan) OR (Ukraine) OR (Georgia) OR (Moldova) OR (Kosovo) OR (Turkey) OR (Serbia) OR (Russia) OR (Kazakhstan) OR (Bulgaria) OR (Belarus) OR (Armenia) OR (Albania))

### Data screening, appraisal and extraction

Screening of eligible studies was done using Microsoft Excel (Microsoft Office 365 ProPlus ; Version 2002) by MA assisted by SK; first by title and abstract and then by full text. Hand-searching of reference lists of eligible studies and relevant reviews was also conducted. For inaccessible articles, personal requests for full texts were made to the authors via email. Any discrepancies arising during the screening stage were resolved through consensus and in consultation with CN. A standardized form was used to extract data relevant to the objectives including study setting, methodology, interventions and outcome measures such as ASMs monitored, purpose and methods of TDM.

The quality of each eligible article was assessed using the Newcastle-Ottawa Scale ( http://www.ohri.ca/programs/clinical_epidemiology/nos_manual.pdf), with a focus on selection- case and control definitions and representativeness (having the outcome of interest); comparability (criteria used to decide on the cases and comparison group and matching), if any; and ascertainment of exposure and non-response rate. This scale has been adapted for appraisal of cohort, cross-sectional and case-control studies and the assessment was done at the study level. Eligible studies were scored out of 10 as per the quality appraisal scale. Those that scored between zero to three were considered poor quality, four to six were rated average quality and studies that scored seven and above were rated good quality. Study selection and data extraction were conducted by MA, assisted by SK who independently reviewed 30% of all eligible full-text articles. Any discrepancies arising during the screening and data extraction were resolved through consensus and in consultation with CN.

### Statistical analysis

Analyses were performed using Stata (StataCorp. 2013. Stata Statistical Software: Release 13. College Station, TX: StataCorp LP). Frequencies between studies were compared using Pearson’s chi-squared test and Fisher’s exact test, when the observations were few. Continuous measures were compared using Man-Whitney U test because most did not follow a Gaussian distribution. Descriptive statistics were reported using measures of central tendency. We had planned to conduct a meta-analysis, but a narrative synthesis was conducted to summarize the outcomes of interest due to significant variability in the reporting of outcomes.

### Protocol registration

The protocol for this review was published on PROSPERO under the registration number CRD42018116937.

## Results

### Search results

**Figure 1.**
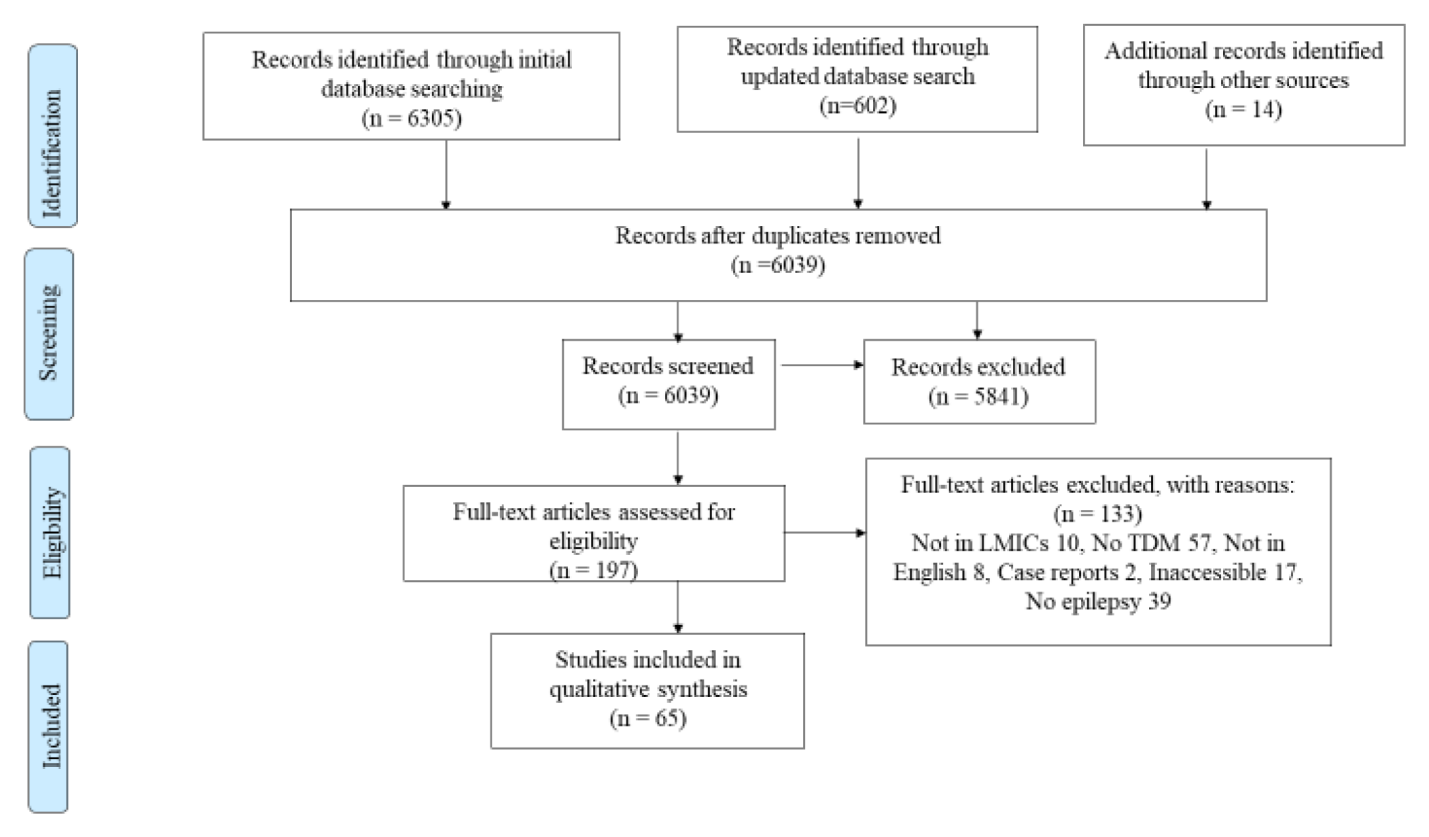
A PRISMA flow diagram used to summarize the number of studies identified, screened, eligible and included and excluded at each stage.

Following a search of four major databases and reference lists of included studies and relevant reviews, 6,039 studies were retrieved. After screening by title and abstract, 198 studies were selected for full text retrieval as shown by the PRISMA flow diagram ( Figure 1). Of the 198 studies, only 65 (32.8%) were eligible for qualitative and quantitative analyses ( Table 2).

**Table 2.** List of included articles.

Author	Year	Study Title
Abaci A, *et al.*	2009	Metabolic alterations during valproic acid treatment: a prospective study
Akin R, *et al.*	1998	Evaluation of bone mineral density in children receiving antiepileptic drugs
Aldemir E, *et al.*	2012	Valproate-associated reproductive hormone abnormalities: do bipolar men have the same risk as epileptic men?
Altunbasak S, *et al.*	1997	Asymptomatic Hyperammonemia in Children Treated With Valproic Acid
Atugonza R, *et al.*	2016	Multiple anti-epileptic drug use in children with epilepsy in Mulago hospital, Uganda: a cross sectional study
Brzakovic B, *et al.*	1997	Cerebrospinal Fluid and Plasma Pharmacokinetics of Phenobarbital after Intravenous Administration to Patients with Status Epilepticus
Brzakovic BB, *et al.*	2012	Impact of age, weight and concomitant treatment on lamotrigine pharmacokinetics
Buchanan N, *et al.*	1977	Serum levels of anticonvulsants and haematological sequelae in Black patients
Cai MW, *et al.*	1993	Free Phenytoin Monitoring in Serum and Saliva of Epileptic Patients in China
Chang Y, *et al.*	2014	Correlation of the UGTIA4 Gene Polymorphism With Serum Concentration and Therapeutic Efficacy of Lamotrigine in Han Chinese of Northern China
Chbili C, *et al.*	2016	Effects of EPHX1 and CYP3A4*22 genetic polymorphisms on carbamazepine metabolism and drug response among Tunisian epileptic patients
Chen Z, *et al.*	2011	Simultaneous determination of valproic acid and 2-propyl-4-pentenoic acid for the prediction of clinical adverse effects in Chinese patients with epilepsy
Cicek NP, *et al.*	2018	The effects of valproate and topiramate use on serum insulin, leptin, neuropeptide y and ghrelin levels in epileptic children
Daci A, *et al.*	2015	Polymorphic Variants of SCN1A and EPHX1 Influence Plasma Carbamazepine Concentration, Metabolism and Pharmacoresistance in a Population of Kosovar Albanian Epileptic Patients
Davies VA, *et al.*	1985	Precursor Prothrombin Status In Patients Receiving Anticonvulsant Drugs
Deda G, *et al.*	2003	Effect of long-term carbamazepine therapy on serum lipids, vitamin B12 and folic acid levels in children
Djordjevic N, *et al.*	2016	CYP1A2 genotype affects carbamazepine pharmacokinetics in children with epilepsy
Dowse and Futter	1991	Outpatient compliance with theophylline and phenytoin therapy
Ecevit C, *et al.*	2004	Effect of carbamazepine and valproate on bone mineral density
Erdal A, *et al.*	2013	The evaluation of sexual functions and sex hormones in male and female epilepsy patients taking valproic acid and carbamazepine monotherapy
Geda G, *et al.*	2002	Serum lipids, vitamin B12 and folic acid levels in children receiving long-term valproate therapy
Gulcebi MI, *et al.*	2018	The effect of serum levetiracetam concentrations on therapeutic response and IL1-beta concentration in patients with epilepsy
Gulcebi MI, *et al.*	2011	The relationship between UGT1A4 polymorphism and serum concentrations of lamotrigine in patients with epilepsy
Hemingway C, *et al.*	1999	The effect of carbamazepine and sodium valproate on the blood and serum values of children from a third-world environment
Hundt HKL, *et al.*	1983	Carbamazepine and its major metabolites in plasma: a summary of eight years of therapeutic drug monitoring
Ibinda F, *et al.*	2017	Magnitude and Factors Associated with Nonadherence to Antiepileptic Drug Treatment in Africa: A cross-sectional multi-site study
Ibinda F, *et al.*	2014	Evaluation of Kilifi Epilepsy Education Programme: A randomized controlled trial
Ilic V, *et al.*	2016	Duration of valproic acid monotherapy correlates with subclinical thyroid dysfunction in children with epilepsy
Incecik F, *et al.*	2007	Effects of valproic acid on hearing in epileptic patients
Jakovljevic MB, *et al.*	2008	Inverse correlation of valproic acid serum concentrations and quality of life in adolescents with epilepsy
Joubert PH, *et al.*	1977	Epilepsy and IgA Deficiency-the Effect of Sodium Valproate
Jovanovic M, *et al.*	2014	Effect of Long-term Topiramate Therapy on Serum Bicarbonate and Potassium Levels in Adult Epileptic Patients
Kurekci AE, *et al.*	1995	Plasma trace element, plasma glutathione peroxidase, and superoxide dismutase levels in epileptic children receiving antiepileptic drug therapy
Lai M	1985	Steady state serum levels of anticonvulsant drugs in Chinese epileptic patients living in Taiwan
Lalic M *et al.*	2009	Lamotrigine and valproate pharmacokinetics interactions in epileptic patients
Leary PM, *et al.*	1989	The prevalence of adverse reactions to anticonvulsants in children with epilepsy
Lu Y, *et al.*	2017	Effects of UGT1A9 genetic polymorphisms on monohydroxylated derivative of oxcarbazepine concentrations and oxcarbazepine monotherapeutic efficacy in Chinese patients with epilepsy
Ma *et al.*	2019	Pharmacist impact on adherence of valproic acid therapy in pediatric patients with epilepsy using active education techniques
Majola MP, *et al.*	2000	Factors influencing phenytoin-induced gingival enlargement
Mbuba CK, *et al.*	2012	Risk factors associated with the epilepsy treatment gap in Kilifi, Kenya: a cross-sectional study
McFayden ML, *et al.*	1990	The relevance of a First-World therapeutic drug monitoring service to the treatment of epilepsy in Third-World conditions
McFayden ML, *et al.*	2007	Therapeutic Drug Monitoring and Clinical Outcomes in Epileptic Egyptian Patients: A gene Polymorphism Perspective Study
Milovanovic DD, *et al.*	2016	The influence of CYP2C8*3 on carbamazepine serum concentrations in epileptic paediatric patients
Milovanovic DD, *et al.*	2015	CYP3A5 polymorphism in Serbian paediatric epileptic patients on carbamazepine treatment
Oner N, *et al.*	2004	Bone mineral metabolism changes in epileptic children receiving valproic acid
Ozkaynakci A, *et al.*	2015	The effect of polymorphic metabolism enzymes on serum phenytoin level
Ozkul Y, *et al.*	2002	Visual functions in epilepsy patients on valproate monotherapy
Placencia M, *et al.*	1993	Antiepileptic drug treatment in a community health care setting in northern Ecuador: a prospective 12-month assessment
Sirmagul B, *et al.*	2012	The effect of combination therapy on the plasma concentrations of traditional antiepileptics: A retrospective study
Smit A, *et al.*	1999	Practical management of therapeutic diphenylhydantoin concentrations in children
Sozuer DT, *et al.*	1997	Serum lipids in epileptic children treated with carbamazepine and valproate
Summers B and Summers RS	1989	Carbamazepine Clearance in Paediatric Epilepsy Patients Influence of Body Mass, Dose, Sex and Co- Medication
Svinarov DA and Pippenger CE	1995	Valproic acid-carbamazepine interaction: Is valproic acid a selective inhibitor of epoxide hydroxylase?
Tanindi S, *et al.*	1996	The platelet aggregation in children with epilepsy receiving valproic acid
Tutanc M, *et al.*	2015	Oxidative Status in Epileptic Children Using Carbamazepine
Ugras and Yakinci	2006	Protein C, protein S and other pro- and anticoagulant activities among epileptic children using sodium valproate
Unay B, *et al.*	2006	Evaluation of renal tubular function in children taking anti-epileptic treatment
Valodia PN	1999	Optimization of phenytoin therapy in adults with epilepsy in the Western Cape, South Africa
Velghe *et al.*	2020	Dried Blood Microsampling-Based Therapeutic Drug Monitoring of Antiepileptic Drugs in Children With Nodding Syndrome and Epilepsy in Uganda and the Democratic Republic of the Congo
Wang P, *et al.*	2015	Effects of CYP3A4/5 and ABCB1 genetic polymorphisms on carbamazepine metabolism and transport in Chinese patients with epilepsy treated with carbamazepine in monotherapy and bitherapy
Wang Q, *et al.*	2015	Effects of UGT1A4 genetic polymorphisms on serum lamotrigine concentrations in Chinese children with epilepsy
Wilmshurst JM, *et al.*	2009	Rescue therapy with high-dose oral phenobarbitone loading for refractory status epilepticus
Yamanturk P, *et al.*	2000	Therapeutic Drug Monitoring in Turkey: Experiences From Istanbul
Yang *et al.*	2019	Comparison of oxcarbazepine efficacy and MHD concentrations relative to age and BMI: Associations among ABCB1, ABCC2, UGT2B7, and SCN2A polymorphisms
Yilmaz Y, *et al.*	2008	The Influence of Valproic Acid Treatment on Hair and Serum Zinc Levels and Serum Biotinidase Activity

### Study characteristics

In terms of WHO regional groupings, the European region contributed a bulk of the studies (53.8%), while the Americas contributed the least (1.5%). Nearly a third (27.7%) of the studies were from the African region while 13.8% were from the Western pacific region ( Table 3). Turkey and South Africa were well-represented with each country contributing 36.9% and 20.2% of the studies, respectively while China and Serbia each contributed 13.0% of the included studies. Those originating from the remaining nine countries each contributed less than 5% of the overall proportion. Majority of the studies were from upper-middle-income countries (87.6%) with studies from low- and lower-middle-income countries contributing less than 10% each ( Table 3).

**Table 3.** Study characteristics.

CHARACTERISTICS	TOTAL NO. OF STUDIES N=65 (%)	TOTAL SAMPLE SIZE N=28921
**Continent** Africa Asia Europe South America	20 (30.7) 9 (13.8) 35 (53.8) 1 (1.5)	7539 2801 18442 139
**WHO Region** African Eastern Mediterranean Western Pacific European Americas	18 (27.7) 2 (3.0) 9 (13.8) 35 (53.8) 1 (1.5)	7271 268 2801 18442 139
**Income group** Low-income Lower-middle-income Upper-middle-income Mixed setting	2 (3.0) 5 (7.6) 57 (87.6) 1 (1.5)	402 1924 25510 1303
**Study design** Case control Cohort Cross-sectional	7 (10.7) 50 (76.9) 8 (12.3)	449 26071 2619
**Study setting** Hospital-based Community-based Both	58 (89.2) 5 (7.6) 2 (3.0)	25757 2874 508
**Urbanization** Rural Urban Both	4 (6.1) 58 (89.2) 3 (4.6)	1813 25455 1871

This review included studies published from 1977 to 2020, with majority (43.0%) being from the last 10 years (2010 to 2020) and cohorts were the most popular study design (76.9%). Fifty-eight (89.2%) of the studies were hospital-based and a similar proportion of the studies was from urban and semi-urban areas ( Table 3). Fifty-three (81.5%) of the included studies were of average quality, 12 (18.4%) studies were classified as high quality and no studies had a score of less than 4 based on the Newcastle-Ottawa scale rating. The age group of participants was not specified in 14 (21.5%) studies but in studies that specified age groups, 41.5% of the studies involved adolescents and children while 36.9% involved adults.

### Anti-seizure medications monitored

First generation ASMs (carbamazepine, phenobarbital, phenytoin and valproic acid) were monitored exclusively in 56 (82.3%) of the studies, while second generation ASMs (including lamotrigine, levetiracetam, oxcarbazepine and topiramate) were monitored in 12 (17.6%) of the included studies. Anticonvulsant benzodiazepines were only monitored in one study ( Table 4). There were 108 instances where TDM of ASMs was reported with some studies reporting monitoring of more than one agent. Valproic acid (30.5%) and carbamazepine (27.7%) contributed to nearly a third of the overall proportion of ASMs monitored with phenytoin and phenobarbital accounting for 17.1% and 13.8%, respectively. The remaining ASMs including lamotrigine, topiramate and oxcarbazepine and benzodiazepines accounted for less than 1.0–5.5%.

**Table 4.** Distribution different ASM classes across WHO regions, continents, income and urbanisation status.

ASM Classification	First generation ASM N (%)	Second generation ASM N (%)	Benzodiazepine N (%)
Overall (N=68)	56 (82.3)	12 (17.6)	1 (1.4)
**WHO Region**			
African (N=19)	18 (94.7)	-	I (5.2)
Americas (N=1)	1 (100.0)	-	-
Eastern Mediterranean (N=2)	2 (100.0)	-	-
European (N=35)	28 (80.0)	5 (14.2)	2 (5.7)
Western Pacific (N=9)	4 (44.4)	5 (55.5)	-
P value	**0.037**	**0.005**	1.000
**Continent**			
Africa (N=21)	20 (95.0)	-	1 (5.0)
Asia (N=9)_	4 (44.4)	4 (44.4)	1 (11.2)
Europe (N=35)	28 (80.0)	5 (14.2)	2 (5.7)
South America (N=1)	1 (100.0)	-	-
P value	**0.016**	**0.010**	0.636
**Income status**			
Low-income (N=3)	3 (100.0)	-	-
Lower-middle-income (N=8)	8 (100.0)	-	-
Upper-middle-income (N= 62)	49 (79.0)	12 (19.3)	1 (1.6)
P value	0.389	0.475	1.000
**Urbanisation**			
Rural (N=7)	7 (100.0)	-	-
Urban (N=65)	52 (80.0)	12 (18.4)	1 (1.5)
P value	0.232	0.262	0.903

There were significant differences across the various WHO regions for TDM of first generation ASMs (p=0.037), with studies from the Americas and Eastern Mediterranean regions not reporting any monitoring of second generation ASMs or anticonvulsant benzodiazepines ( Table 4). A similar trend for first generation ASMs was observed in the various continents with significant differences in the proportions (p=0.016). However, studies from Asia had an equal distribution (44.4%) of studies reporting monitoring of first and second generation ASMs ( Table 4). There were no significant differences in monitoring of either first or second generation ASMs or benzodiazepines across studies from different income levels or urban and rural settings.

### Purpose of Therapeutic Drug Monitoring (TDM)

Overall, there were 77 instances where the reasons for conducting TDM were reported with some studies reporting multiple purposes for TDM. Monitoring to assess side effects constituted nearly half (42.8%) of the reported instances while monitoring with the aim of evaluating compliance and to examine effect of genetics on ASM levels accounted for 12 (15.5%) and 11 (14.2%) of the instances. Other reasons for TDM that were reported accounted 2.5 to 10.3% of the instances, including monitoring to assess clinical efficacy, to determine drug interactions or for the purpose of dose titration ( Table 5).

**Table 5.** Distribution of various purposes of TDM across WHO regions, continents, income group and urbanization status.

Purpose of TDM	Side Effects N (%)	Adherence N (%)	Clinical Efficacy N (%)	Drug Interactions N (%)	Dose Titration N (%)	Method Validation N (%)	Genetic Variations N (%)
**Overall** (N=77)	33 (42.8)	7 (9.0)	12 (15.5)	8 (10.3)	4 (5.1)	2 (2.5)	11 (14.2)
**Region**							
Americas (N=1)	-	-	1 (100.0)	-	-	-	-
Western Pacific (N=16)	1 (6.2)	1 (6.2)	3 (18.7)	3 (18.7)	1 (6.2)	2 (12.5)	5 (31.2)
Europe (N=35)	22 (62.8)	-	3 (8.5)	4 (11.4)	1 (2.8)	-	5 (14.2)
Eastern Mediterranean (N=3)	-	-	2 (66.6)	-	-	-	1 (33.3)
African (N=23)	10 (43.4)	6 (26.0)	3 (13.0)	1 (4.3)	2 (8.6)	1 (4.3)	-
P value	**>0.05**	**0.012**	**0.023**	0.528	0.635	0.215	**0.025**
**Continent**							
South America (N=1)	-	-	1 (100.0)	-	-	-	-
Asia (N=17)	2 (11.7)	1 (5.8)	3 (17.6)	3 (17.6)	1 (5.8)	2 (11.7)	5 (29.4)
Europe (N=35)	22 (62.8)	-	3 (8.5)	4 (11.4)	1 (2.8)	-	5 (14.2)
Africa (N=25)	10 (40.0)	6 (24.0)	5 (20.0)	1 (4.0)	2 (8.0)	1 (4.0)	-
P value	**0.002**	**0.007**	0.108	0.416	0.823	0.122	**0.020**
**Income**							
Low-income (N=4)	1 (25.0)	1 (25.0)	1 (25.0)	-	-	1 (25.0)	-
Lower-middle- income (N=5)	-	1 (20.0)	2 (40.0)	-	-	-	2 (40.0)
Upper-middle- income (N=69)	32 (46.3)	5 (7.2)	9 (13.0)	8 (11.5)	4 (5.7)	2 (2.8)	9 (13.0)
P value	0.097	0.183	0.184	1.000	1.000	0.157	0.226
**Urbanization**							
Urban (N=74)	33 (44.5)	5 (6.7)	11 (14.8)	8 (10.8)	4 (5.4)	2 (2.7)	11 (14.8)
Rural (N=7)	1 (14.2)	3 (42.8)	2 (28.5)	-	-	1 (14.2)	-
P value	0.123	**0.019**	0.372	0.469	0.692	0.240	0.345

There were significant differences across the various WHO regions of monitoring to assess unwanted ASM side effects (p<0.05) accounting for 62.8% and 43.4% of the instances in the European and African regions, respectively ( Table 5). Across the regions, there were also significant differences in TDM conducted to examine the effect of genetic variations on ASM levels (p= 0.025), making up a third (31.2% and 33.3%) of the proportions in the Western Pacific and Eastern Mediterranean regions. In addition, there were significant differences across the regions of TDM to assess adherence (p=0.012) and clinical efficacy (p=0.023) and a similar trend was observed across the continents. There were no significant differences in purposes of TDM across various income levels ( Table 5). A similar trend was observed when urban and rural areas were compared except for TDM conducted to assess compliance which accounted for nearly half (42.8%) of the TDM in rural areas but only 6.7% in urban areas (p=0.019).

### Methods of TDM

Twelve different TDM techniques were reported in 50 (76.3%) of the included studies with some studies reporting more than one method (53 instances). Fluorescence polarization immunoassay (FPIA) was reported in nearly half (41.5%) of the instances ( Table 6), while High Performance Liquid Chromatography (HPLC) and HPLC with ultraviolet detection (HPLC-UV) were used in 9 (16.9%) and 5 (9.4%) of the instances. The remaining techniques including Liquid Chromatography-Mass Spectrometry (LC-MS) and Gas Chromatography (GC) accounted for 1.8 to 5.6% of the overall proportion ( Table 6).

**Table 6.** Various TDM methods and estimated commercial cost of equipment.

Method of TDM (N = 50 studies)	Total N= 53 (%)	Estimated Cost of Equipment
Auto-analyzer	3 (5.6)	$14,000
Chemiluminescent Immunoassay	2 (3.7)	Unknown
Enzyme Multiplied Immunoassay Technique	3 (5.6)	$1600 (Cobas), $11,500 (Syva)
Fluorescence Polarization Immunoassay	22 (41.5)	$1000 (TDx)
Gas Chromatography	2 (3.7)	$79,500
High Performance Liquid Chromatography (HPLC)	9 (16.9)	$21,500
HPLC with fluorescence detection	1 (1.8)	$1799.99
HPLC with Ultraviolet detection (HPLC-UV)	5 (9.4)	$2,695
Liquid Chromatography-Mass Spectrometry LC-MS	3 (5.6)	$149,000
Reversed phase HPLC with UV detection	1 (1.8)	$1100
Thin Layer Chromatography (TLC)	1 (1.8)	Unknown
TLC with fluorescence detection	1 (1.8)	Unknown

FPIA was the most common method of TDM in the African (64.2%) and European regions (42.3%) with a trend toward significance for differences across the regions (p=0.075). HPLC was popular in studies from the Eastern Mediterranean (50.0%) and Western Pacific (36.3%) regions with a significant difference across the regions (p=0.035). A similar trend was observed across the continents, but with no significant differences while the TDM method used in the single study from South America (Americas region) was not reported. The trend remained same across different income levels and in urban and rural areas with FPIA being the most popular TDM method in low- (50.0%), lower-middle- (60.0%) and upper-middle-income (41.3%) countries and urban (40.0%) and rural (75.0%) areas. There were no significant differences across the studies across different income levels or when comparing studies from urban and rural areas.

The overall cost of the various techniques was unspecified in all the included studies, therefore, the cost of purchase of equipment was used to allude to the likely cost per test. LC-MS equipment was the most expensive based on commercially quoted prices, costing about $149,000 (TSQ Quantum Access from Thermo-Fischer), while FPIA equipment (TDx from Abbot Technologies) was the least expensive, costing about $1,000 ( Table 6).

### Assay validation and quality control

Fourteen (23%) of the included studies described validation of the various assays used in TDM. Nine (64%) of these studies reported either overall coefficients of variation (CV), intra- and interassay CV, percentage accuracy, percentage precision and intra- and interday measures of accuracy including reproducibility and relative standard deviation. This significant variability in reported outcomes made it difficult to pool these outcomes.

Among studies that reported intra- and interassay CVs, one study using LC-MS/MS to assay phenobarbital, valproic acid and carbamazepine in dried blood spots reported 3.8% and 4.1% as inter- and intraassay CV, respectively. Accuracy and precision in this study was reported as below 13% and 10%, respectively ( Velghe *et al.*, 2020). Two studies using FPIA to assay phenytoin reported the mean intra- and interassay CVs were 4.2% (interquartile range [IQR] 2.1-6.4) and 4.4% (IQR 3.4-5.4) respectively ( Cai *et al.*, 1993; Smit *et al.*, 1999).

Among studies that reported intra- and interday measures, one study that used both HPLC and GC to assay valproic acid reported the intra- and interday reproducibility ranging from 1.54 to 5.14% and 1.59 to 4.97%, respectively for HPLC with a precision within 5.1% and from 0.59 to 2.04% and 2.67 to 5.2%, respectively for GC with the precision within 5.5% ( Ma *et al.*, 2019). A different study that used HPLC to assay 10-hydoroxycarbazepine reported the intra and interday precision as ranging from 0.58 to 16.67% ( Yang *et al.*, 2019) while another study that used HPLC-UV to assay carbamazepine (CBZ) reported the intra- and inter-day precision of CBZ and carbamazepine-epoxide (CBZ-E) as ranging from 0.56% to 6.14% ( Wang *et al.*, 2015a).

The inter- and intraday relative standard deviations of lamotrigine assays using HPLC reported in a different study were lower than 15% for all measured analytes while for valproic acid, the assay using GC had a coefficient of variation (C.V.) lower than 4% ( Wang *et al.*, 2015b). One study reported the inter- and intraday CV for lamotrigine assayed using HPLC as 6.57 ± 2.01% and 4.95 ± 1.87% precision ( Brzaković *et al.*, 2012) while another reported similar CV being less than 8% and less than 12% respectively, for phenobarbital in both cerebrospinal fluid and plasma assayed using reversed-phase HPLC-UV ( Brzaković *et al.*, 1997). In terms of quality control, duplicate determinations were the most commonly used quality control methods and were reported in four (28%) of these studies, two studies on lamotrigine and two on phenytoin.

### Reference ASM ranges used during TDM

Eighteen (27.6%) of the included studies reported the ASM reference ranges used during TDM. Majority of these studies were from the African (38.8%) and European regions (30.0%) with the other regions contributing between 5 to 16.6% of the proportion. Overall, uniform reference ranges as defined by ILAE were used for phenytoin (10.0-20.0µg/ml), valproic acid (50.0-100.0µg/ml), lamotrigine (3.0-14.0µg/ml) and phenobarbital (10.0-40.0µg/ml) while the median reference range used for carbamazepine (4.0-11.8µg/ml) varied from the set limits. Across the regions, similar findings were observed for carbamazepine while phenobarbital median reference ranges also varied ( Table 7).

**Table 7.** Median reference ranges used during TDM across the regions.

ASM	Carbamazepine		Phenobarbital		Phenytoin		Valproic acid		Lamotrigine	
Reference range	Lower limit (IQR)	Upper limit (IQR)	Lower limit (IQR)	Upper limit (IQR)	Lower limit (IQR)	Upper limit (IQR)	Lower limit (IQR)	Upper limit (IQR)	Lower limit (IQR)	Upper limit (IQR)
Overall N=18	4.0 (4.0-4.6)	11.81 (10.0-12.0)	10.0 (9.2-10.0)	40.0 (32.4-40.0)	10.0 (10.0-10.0)	20.0 (20.0-20.0)	50 (50.0-50.2)	100.0 (100.0-100.0)	-	-
**WHO Region**										
African N=7	4.0 (4.0-4.0)	12 (11.8-12.0)	10 (9.2-10.0)	40 (25.5-40.0)	10.0 (10.0-10.0)	20.0 (20.0-20.0)	50 (50.0-50.2)	100.0 (100.0-100.0)	-	
European N=6	4.0 (4.0-4.0)	10.0 (10.0-10.0)	12.5 (10.0-15.0)	40.0 (40.0-40.0)	10.0 (10.0-10.0)	20.0 (20.0-20.0)	50.0 (50.0-50.0)	100.0 (100.0-100.0)	3.0 (3.0-3.0)	14.0 (14.0-14.0)
Western Pacific N=3	-	-	-	-	10.0 (10.0-10.0)	20.0 (20.0-20.0)	50.0 (50.0-50.0)	100.0 (100.0-100.0)	-	-
Eastern Mediterranean N=1	-	-	-	-	10.0 (10.0-10.0)	20.0 (20.0-20.0)				
Americas N=1	4.6 (4.6-4.6	11.6 (11.6-11.6)	9.2 (9.2-9.2)	39.4 (39.4-39.4)	-	-	-	-	-	-

## Discussion

To the best of our knowledge, this is the first systematic review to summarize evidence on therapeutic drug monitoring of ASMs in LMICs, including the methods used, reference ranges and purposes of conducting TDM. Majority of TDM was conducted in Europe compared to the rest of the world and in upper middle-income countries compared to low- and lower-middle-income countries, perhaps because of logistical burden involved. TDM for first generation ASMs was commonly reported overall while and monitoring to assess unwanted ASM-related side effects was the most popular reason for conducting TDM. FPIA was the most common method used for TDM, consistent with its the lowest commercial quote compared to other equipment. Assay validation and quality control were reported in a small proportion of the studies with variability in the reporting of outcomes. Majority of the studies used the International League Against Epilepsy (ILAE)-recommended reference ranges ( Patsalos *et al.*, 2008) during TDM.

### TDM by geographic regions and economic blocks

TDM appeared to be influenced by economic status and infrastructural status. For example, more than half (53%) of TDM was in Europe (as a WHO region or continent), where countries can afford TDM apparatus and machines compared to the rest of the world. Similarly, Turkey and Serbia, which are upper middle-income countries from Europe, were leading in TDM compared to other individual countries. Africa contributed a substantial proportion of TDM (27%), but economically developed countries in Africa were over-represented, for instance, South Africa, which was leading in Africa, contributed nearly 20% of all TDM. Indeed, over 87% of all TDM was in upper-middle-income countries, which are wealthier than low- and lower-middle-income countries. Therefore, there is need to increase investment on TDM in LMICs. 

### Purpose of TDM

TDM was mostly conducted to assess ASM side effects and toxicities, which are common with first generation ASMs ( Nimaga *et al.*, 2002). ASM-related toxicities were more likely to be monitored in Africa and Europe which contributed the bulk of all included studies. TDM to examine the effect of genetic variations on ASM levels was more common in the Western Pacific and Eastern Mediterranean regions, where there is capacity to screen for genetic polymorphisms that are very common in these populations ( Chang *et al.*, 2014); ( Chbili *et al.*, 2016). TDM to assess ASM compliance contributed a large proportion in rural areas and could be attributed to poor health-seeking behaviour observed in these settings, which can affect response to treatment. For instance, a study in rural Kilifi estimated nonadherence based on optimal levels in this setting at nearly 80% ( Ibinda *et al.*, 2017). Nonadherence has been associated with symptomatic epilepsy and long duration epilepsy lasting longer than 10 years ( Mbuba *et al.*, 2012), which may be proxies for refractory epilepsy that is yet to be fully characterized in Africa.

### Methods and techniques used in TDM

FPIA was the most common technique used for TDM overall and in Africa and Europe compared to others such as HPLC. In Africa, this could be due to the relatively low cost of equipment (TDx system) based on commercially quoted prices and low sample volumes ( Kang *et al.*, 2012). Surprisingly, FPIA was equally popular in Europe, implying possible knowledge exchange and collaboration between the continents. HPLC, which is slightly more expensive in terms of equipment, was popular in Asian countries probably informed by efficiency or production and assemblage of these machines in those settings. The WHO recommends an estimation of cost per test based on equipment, reagents and consumables, personnel, facility and quality management but only commercially quoted equipment prices were available for comparison ( World Health Organization, 2019). For the studies that reported assay validation and quality control during TDM, authors reported compliance with either international or national bioanalytical guidelines, but this could not be validated.

### Recommended therapeutic ranges of ASMs during TDM

In 2008, a best practice guideline was developed by ILAE ( Patsalos *et al.*, 2008) to guide therapeutic drug monitoring of anti-seizure medications and interpretation of the ASM levels. Only a very small proportion of the studies reported the reference range used during the assays, mostly from Africa and Europe (the regions that formed the bulk of the included studies). Among the studies, all the reported reference ranges were within the ILAE recommendations except for variations in ranges used for phenobarbital and carbamazepine. These variations could be attribute to ranges used in three studies published before the publication of the guidelines in 2008. Notably, ILAE recommends use of the reference ranges as a benchmark upon which individual concentrations should be established to achieve the best clinical outcomes, and this should be emphasized in future studies in LMIC.

### Strengths and limitations

This review outlines findings on TDM that are generalizable to LMICs, including middle-income countries in Europe, which may have easily been overlooked. The review followed standard procedures such as a PRISMA flow-diagram used to clearly elucidate the inclusion and exclusion criteria. For references ranges used during TDM of the various agents, we used the recommended ILAE guidelines to determine appropriateness.

However, this review has several limitations. Significant between-study variation was observed across the outcomes of interest and can be attributed to different study designs, methods of TDM, sample sizes, types of ASMs measured and the different populations with varying ethnicities in the included studies. Majority of the studies were from Eastern Europe, specifically Turkey and Serbia, which may not be representative of the poorer continents. In Africa. majority of the studies were from South Africa, an upper-middle-income country, which may not represent most low- and lower middle-income groups. Some studies were based on very small sample sizes that may not offer acceptable precision for comparing the outcomes of interest. The estimated cost of the various TDM methods was extrapolated from the cost of equipment as data required for laboratory costing was not reported in any of the studies. Finally, it is possible there was some publication bias due to failure to report important aspects by some authors such as the reference ranges used during TDM which form the basis of monitoring.

## Conclusions and public health implications

There is evidence of TDM in LMICs, especially in upper-middle-income countries, possibly due to availability of resources and technical expertise. First generation ASMs were the most commonly monitored and TDM was mostly conducted for the purpose of assessing unwanted side effects and toxicities related to ASMs. FPIA was the most common method of TDM and likely to be the most affordable.

There is need for TDM of second generation ASMs, which are in use in some LMIC including the African region with little to no monitoring. Training of primary health care providers on appropriate prescription of ASMs in epilepsy should be prioritised. Routine TDM should be encouraged specifically in low-income countries where first generation ASMs, associated with unwanted side effects and toxicities, are mostly prescribed.

## Data availability

### Underlying data

All data underlying the results (except for individual participant data) are available as part of the article and no additional source data are required. For data pertaining to the individual participants in the included articles, this is deemed to be licensed under the authors of the primary studies.

### Reporting guidelines

Havard Dataverse: PRISMA checklist for ‘Therapeutic monitoring of anti-seizure medications in low- and middle-income countries: a systematic review’, https://doi.org/10.7910/DVN/JMBK4R ( Odhiambo **et al.*,* 2021)

Data are available under the terms of the Creative Commons Attribution 4.0 International license (CC BY 4.0).
