# Supplementary material for: Therapeutic monitoring of anti-seizure medications in low- and middle-income countries: a systematic review
Source: Wellcome Open Res. 2024 Jan 31;6:92. Originally published 2021 Apr 29. [Version 3] doi: 10.12688/wellcomeopenres.16749.3 (PMC10349269; doi:10.12688/wellcomeopenres.16749.3)
Supplement: Peer review history

# Reviewer report: Reviewer response for version 2

**DOI:** 10.21956/wellcomeopenres.21575.r58288

**Published:** 2023-07-14

**Authors:** Bridget MacDonald

**Affiliations:** 1 Croydon University Hospital, Thornton Heath, CR7 7YE, UK

I think this article is fine now and deserves to be indexed.

 I retain my concerns about what appears to be a belief that it's good to monitor drugs to see if there are side effects, as opposed to clinically assessing patients adequately and listening to them.

 There is no comment on whether tests are done more if the system for reimbursing doctors rewards doctors to do more tests. I think this does influence therapeutic drug monitoring in some countries.

Are the rationale for, and objectives of, the Systematic Review clearly stated?

No

Is the statistical analysis and its interpretation appropriate?

Partly

Are sufficient details of the methods and analysis provided to allow replication by others?

Yes

Are the conclusions drawn adequately supported by the results presented in the review?

Partly

Reviewer Expertise:

Epidemiology, epilepsy, general neurology

I confirm that I have read this submission and believe that I have an appropriate level of expertise to confirm that it is of an acceptable scientific standard.

# Reviewer report: Reviewer response for version 1

**DOI:** 10.21956/wellcomeopenres.18471.r48086

**Published:** 2022-01-25

**Authors:** Bridget MacDonald

**Affiliations:** 1 Croydon University Hospital, Thornton Heath, CR7 7YE, UK

Thank you for doing this study which is interesting and has value. I am commenting from the perspective of a neurologist with an interest in epilepsy and epidemiology with some experience of advising colleagues in low income countries. I am not in any way an expert in how therapeutic drug monitoring is carried out although I may use its results in clinical practice. Hence I will not comment on that aspect at all.

 There are, for me, several issues that need to be clarified.

 Most importantly this study cannot be read as a study of where and how much TDM is used in different places of the world. It is an account of who has written articles about TDM. It would be wrong to conflate these two separate things. The article should be altered to reflect this.

 More specific issues: (I leave the citation from the paper in italics, my comments in normal text)

- *For instance, phenobarbital is associated with adverse effects including risk of withdrawal seizures, hyperactivity in children, risk of drug abuse and suicidal ideation ( Nimaga et al., 2002), but it is the only ASM that is easily available and affordable in many LMICs ( Chin, 2012). These adverse effects require monitoring but TDM is rarely done in LMICs due to limited infrastructure, resources and lack of technical expertise to operate equipment for mass spectrometry and immunoassays ( Tomson et al., 2007).*
  This paragraph is somewhat cherry picking – using a citation to support the notion that phenobarbital is “bad”, when some evidence is contrary to that view e.g. when Deb Pal (Randomised controlled trial to assess acceptability of phenobarbital for childhood epilepsy in rural India. Lancet Volume 351, Issue 9095, 3 January 1998, Pages 19-23) ^1^ looked into this in a well designed study in poor village children in India the outcomes are far more nuanced.
- *Routine monitoring allows the prescriber to check treatment efficacy while identifying adverse effects and drug interactions. Drug interactions are often complicated by pharmacogenetics, medication for comorbidities and patient adherence ( Knezevic & Marzinke, 2018).*
  It seems to me that although this view is often heard what one needs is to discuss whether there is evidence demonstrating that therapeutic drug monitoring achieves the aim of better therapeutic effect with fewer side-effects better than good clinical practice without TDM. UK’s NICE guidelines, for example, does not advocate their use except under a limited number of circumstances because there is little evidence to support it. There is marked variance in the use of TDM across Europe which is better explained by tradition than research.
- In Methods – what did you do to ascertain that articles written in languages other than English were picked up and then analysed? All search terms were in English and only 8 were identified as eligible but not analysed because they were not in English. One might, for example, expect American literature to have Spanish and Portuguese articles. Overall the non-English group seems low to me and so more comment on this needs to be made
- *There is a need for more investment in comprehensive TDM in LMICs that incorporates dose titration of ASM using pharmacokinetics and pharmacodynamics modelling, for both first generation and second generation ASMs*
  I am unsure that there is evidence that TBM achieves better clinical outcomes, it could be argued that the shortfall in treatment for people with epilepsy in resource poor settings would be made worse if unnecessary and expensive monitoring were advocated. It seems there is a better rationale for monitoring when introducing (novel) medications to new populations where the risk of marked differences in drug metabolism might be suspected (be that due to diet or genetics) as part of an "introduction of a new drug to new population" study rather than a monitoring system.

In all, this article is interesting and well written but I find the framing of the study not quite right. Additionally, it purports to measure something it does not. The text could be altered to reflect this without altering the value of the data and work that has gone into it.

Are the rationale for, and objectives of, the Systematic Review clearly stated?

No

Is the statistical analysis and its interpretation appropriate?

Partly

Are sufficient details of the methods and analysis provided to allow replication by others?

Yes

Are the conclusions drawn adequately supported by the results presented in the review?

Partly

Reviewer Expertise:

epidemiology, epilepsy, general neurology

I confirm that I have read this submission and believe that I have an appropriate level of expertise to confirm that it is of an acceptable scientific standard, however I have significant reservations, as outlined above.

## References

[1] : Randomised controlled trial to assess acceptability of phenobarbital for childhood epilepsy in rural India. *The Lancet* .1998;351(9095) : 10.1016/S0140-6736(97)06250-8 19-23 10.1016/S0140-6736(97)06250-8 9433424

# Response

**Published:** 2023-05-09

**Authors:** Mercy Odhiambo

**Affiliations:** Kenya Medical Research Institute, Kenya

Thank you for taking the time to review our article. Please see below our responses to your comments. Most importantly this study cannot be read as a study of where and how much TDM is used in different places of the world. It is an account of who has written articles about TDM. It would be wrong to conflate these two separate things. The article should be altered to reflect this.

**Response: Thank you for your comment. We think that lack of published articles may not necessarily mean that TDM is not being conducted in these settings as TDM may be conducted in clinics and hospitals but not published if not conducted systematically or in the context of research. However, we have reworded the objective to emphasise this that this review was conducted to examine available evidence on TDM, purposes, and methods and ASM reference ranges used during monitoring in these settings. **  

 More specific issues: (I leave the citation from the paper in italics, my comments in normal text) *For instance, phenobarbital is associated with adverse effects including risk of withdrawal seizures, hyperactivity in children, risk of drug abuse and suicidal ideation (Nimaga et al., 2002), but it is the only ASM that is easily available and affordable in many LMICs (Chin, 2012). These adverse effects require monitoring but TDM is rarely done in LMICs due to limited infrastructure, resources and lack of technical expertise to operate equipment for mass spectrometry and immunoassays (Tomson et al., 2007).* This paragraph is somewhat cherry picking – using a citation to support the notion that phenobarbital is “bad”, when some evidence is contrary to that view e.g. when Deb Pal (Randomised controlled trial to assess acceptability of phenobarbital for childhood epilepsy in rural India. Lancet Volume 351, Issue 9095, 3 January 1998, Pages 19-23)1 looked into this in a well designed study in poor village children in India the outcomes are far more nuanced.****

**Response: Thank you for the observation. Indeed, some studies show benefit while other show some adverse effects of most anti-seizure medication in use. We have re-written this section to comment on adverse events related to anti-seizure medications in general such as feelings of tiredness, stomach upset, dizziness, blurred vision, cognitive and behavioural problems (Kwan et al., 2013; Mutanana, Tsvere, & Chiweshe, 2020) as this remains a concern and justification for TDM.**

*Routine monitoring allows the prescriber to check treatment efficacy while identifying adverse effects and drug interactions. Drug interactions are often complicated by pharmacogenetics, medication for comorbidities and patient adherence (Knezevic & Marzinke, 2018).* It seems to me that although this view is often heard what one needs is to discuss whether there is evidence demonstrating that therapeutic drug monitoring achieves the aim of better therapeutic effect with fewer side-effects better than good clinical practice without TDM. UK’s NICE guidelines, for example, does not advocate their use except under a limited number of circumstances because there is little evidence to support it. There is marked variance in the use of TDM across Europe which is better explained by tradition than research.

**Response: Thank you for the observation.  We have elaborated on the benefit of monitoring; emphasising the highly variable pharmacokinetic profiles of these ASM. For instance, phenytoin displays zero-order pharmacokinetics meaning it can easily accumulate and cause toxicity if not monitored while carbamazepine has a narrow therapeutic index meaning a slight change in its dose may be the difference between sub-clinical response and toxicity. These variations may be empirically assessed clinically but can only be ascertained through TDM.**

 In Methods – what did you do to ascertain that articles written in languages other than English were picked up and then analysed? All search terms were in English and only 8 were identified as eligible but not analysed because they were not in English. One might, for example, expect American literature to have Spanish and Portuguese articles. Overall the non-English group seems low to me and so more comment on this needs to be made

**Response: This is a good observation. Even though our entire search strategy was in English, we postulate that most non-English articles will have English translated titles and abstracts, so these would be picked up during the search. We have added this explanation in the search strategy section and also noted this as a limitation.**

*There is a need for more investment in comprehensive TDM in LMICs that incorporates dose titration of ASM using pharmacokinetics and pharmacodynamics modelling, for both first generation and second generation ASMs* I am unsure that there is evidence that TBM achieves better clinical outcomes, it could be argued that the shortfall in treatment for people with epilepsy in resource poor settings would be made worse if unnecessary and expensive monitoring were advocated. It seems there is a better rationale for monitoring when introducing (novel) medications to new populations where the risk of marked differences in drug metabolism might be suspected (be that due to diet or genetics) as part of an "introduction of a new drug to new population" study rather than a monitoring system.

**Response: Thank you for this observation. Indeed, monitoring ca be cost-intensive, and we propose introducing monitoring using a targeted approach. It would be useful in establishing the efficacy and side effect profile of new drugs as suggested. Additionally, in cases of suspected drug-related adverse effects for patients on polytherapy, for instance, it would be difficult to identify the offending drug at first glance but TDM would offer a fairly quick resolution. We have re-framed the conclusion to reflect situations where TDM would be most useful in low-income settings and play a role in improving patient outcomes.**

# Reviewer report: Reviewer response for version 1

**DOI:** 10.21956/wellcomeopenres.18471.r43659

**Published:** 2021-05-19

**Authors:** Wondim Ayenew

**Affiliations:** 1 Department of Pharmaceutics, College of Health Science, School of Pharmacy, University of Gondar, Gondar, Ethiopia

Generally, the title and their work are interesting but I have some comments that will be seen by authors. 

1. Title: There is a difference between seizure and epilepsy but you write antiseizure medication in your title but you are talking about epilepsy in your background and other sections so please revise your title and the terms antiseizure medications in your manuscript. 
2. Abstract section: Method: Please add the search period and the software used for your analysis
3. In your main manuscript, you put the protocol registration before the result section. because you followed PRISMA guidelines, the protocol registration should be written just after method and before inclusion and exclusion criteria.
4. The quality of observational studies is assessed based on the Newcastle Ottawa quality assessment scale adapted for crossectional studies. So it is advisable to use that adapted assessment scale rather than adapting by yourself. 
5. Where are your outcome measurements?
6. In your strength and limitation part of paragraph 2 line 16, you mentioned as there is publication bias. Where is the publication bias and how you assessed it? 

Are the rationale for, and objectives of, the Systematic Review clearly stated?

Yes

Is the statistical analysis and its interpretation appropriate?

Yes

Are sufficient details of the methods and analysis provided to allow replication by others?

Yes

Are the conclusions drawn adequately supported by the results presented in the review?

Yes

Reviewer Expertise:

cancer

I confirm that I have read this submission and believe that I have an appropriate level of expertise to confirm that it is of an acceptable scientific standard.

# Response

**Published:** 2023-05-09

**Authors:** Mercy Odhiambo

**Affiliations:** Kenya Medical Research Institute, Kenya

Thank you for taking the time to review our article. Please see below our responses to your comments.

 Title: There is a difference between seizure and epilepsy but you write antiseizure medication in your title but you are talking about epilepsy in your background and other sections so please revise your title and the terms antiseizure medications in your manuscript.

**Response: anti-seizure medications (ASM) is the current recommended term, having been changed from the previously used term; anti-epileptic drugs. The reason for this change is because these ASM are known to suppress the seizure activity and do not affect the underlying disease i.e. that is epilepsy. This will allow communication between patients and physicians that symptomatic treatments such as ASM do not cure the disease and have to be taken at regular intervals to prevent recurrent seizures.**

 Abstract section: Method: Please add the search period and the software used for your analysis

**Response: We have added the search period (initially on 8th November 2018 and updated on 31st December 2020) and the software used for analysis (Stata 13) in the abstract.**

 In your main manuscript, you put the protocol registration before the result section. because you followed PRISMA guidelines, the protocol registration should be written just after method and before inclusion and exclusion criteria.

**Response: We have moved the protocol registration section to immediately after the title of the methods section.**

 The quality of observational studies is assessed based on the Newcastle Ottawa quality assessment scale adapted for cross sectional studies. So, it is advisable to use that adapted assessment scale rather than adapting by yourself.

**Response: We used all the quality appraisal tools as obtained from the source and we did not adapt any by ourselves, but rather applied it to the aspects available in the searched studies. We have re-phrased the wording to reflect that** **we used the published NOS that has been adapted for cross-sectional and case-control studies.**

 Where are your outcome measurements?

**Response: Thank you for this observation. We extracted data on the regional distribution of the included articles, type of ASM used, method of conducting TDM, validation procedures and references ranges. We have added the outcome measurements in the methods and they include the proportion of TDM by country, continent, WHO region and income level as a percentage, the proportions of ASM monitored by country, continent, WHO region, income level and classification as a percentage and the mean and median reference ranges used during monitoring by ASM.**

 In your strength and limitation part of paragraph 2 line 16, you mentioned that there is publication bias. Where is the publication bias and how you assessed it? **Response: We postulate that they may be publication bias due to lack of publication of negative results as well as other biases due to methodological differences that we could not evaluate in the present analysis. We have added this statement to the text to clarify the same.**
